# Humanization and Characterization of an Anti-Human TNF-α Murine Monoclonal Antibody

**DOI:** 10.1371/journal.pone.0016373

**Published:** 2011-01-31

**Authors:** Wei-Chun Chiu, Ya-Ping Lai, Min-Yuan Chou

**Affiliations:** Biomedical Technology and Device Research Laboratories, Industrial Technology Research Institute, Hsinchu, Taiwan; University of California, Merced, United States of America

## Abstract

A murine monoclonal antibody, m357, showing the highly neutralizing activities for human tumor necrosis factor (TNF-α) was chosen to be humanized by a variable domain resurfacing approach. The non-conserved surface residues in the framework regions of both the heavy and light chain variable regions were identified *via* a molecular modeling of m357 built by computer-assisted homology modeling. By replacing these critical surface residues with the human counterparts, a humanized version, h357, was generated. The humanized h357 IgG_1_ was then stably expressed in a mammalian cell line and the purified antibody maintained the high antigen binding affinity as compared with the parental m357 based on a soluble TNF-α neutralization bioassay. Furthermore, h357 IgG_1_ possesses the ability to mediate antibody-dependent cell-mediated cytotoxicity and complement dependent cytotoxicity upon binding to cells bearing the transmembrane form of TNF-α. In a mouse model of collagen antibody-induced arthritis, h357 IgG significantly inhibited disease progression by intra-peritoneal injection of 50 µg/mouse once-daily for 9 consecutive days. These results provided a basis for the development of h357 IgG as therapeutic use.

## Introduction

Tumor necrosis factor (TNF-α) is a pro-inflammatory cytokine produced primarily by cells of the immune system, including macrophages and monocytes. TNF-α is present as a homotrimeric protein in which each subunit is initially translated as a 26 kDa transmembrane precursor protein. After being cleaved at a site proximal to the transmembrane domain of TNF-α by TNF-α converting enzyme, a soluble trimeric form of TNF-α is released and exerts its activity by binding to two structurally distinct type I and type II TNF receptors (TNFRI and TNFRII) on effector cells. The transmembrane form of TNF-α is also known as its unique biologic functions, such as cytotoxic activity and polyclonal B cell activation, in a cell-to-cell contact manner [1]. TNF-α has been proved to have certain effects on autoimmune processes and has become a key therapeutic target for many autoimmune diseases [2]. So far, some anti-TNF-α agents, like etanercept, adalimumab and infliximab were approved by the Food and Drug Administration, and all have the capability to neutralize soluble form of TNF-α effectively as a major pharmacological mechanism of action. However, the binding effects of these antagonists on the transmembrane form of TNF-α are different, which may cause different results on clinical diseases [3]. For instance, etanercept is not clinically effective for the pathogenesis of granulomatous diseases, in which the transmembrane form of TNF-α may play a critical role [1]. Therefore, whether or not anti-TNF-α agents can bind to the transmembrane form of TNF-α is prerequisite to trigger antibody dependent cell mediated cytotoxicity (ADCC), complement dependent cytotoxicity (CDC), apoptotic and outside-to-inside signaling mechanisms.

The major impediment of the murine monoclonal antibody in clinical practice is that it may elicit human anti-murine antibody (HAMA) response in patients [4], [5], [6]. Hence, to improve the efficiency in clinical use, genetic engineering technology has been employed to replace the murine content with the amino acid residues of human counterparts, and to reduce the possibility of inducing immunogenicity in patients. An ideal antibody humanization should be capable of maintaining the specificity and affinity toward the antigen and reduces the immunogenicity as much as possible. So far, many approaches have been used for antibody humanization, such as chimeric antibodies, which consists of murine antigen-binding variable regions fused genetically to human antibody constant regions, is the earliest attempt to reduce immunogenicity [7]. However, chimeric antibodies still generate undesirable anti-variable region response [8]. Complementarity determining region (CDR)-grafting is another approach involving the transfer of the CDRs from a rodent antibody to the Fv frameworks (FRs) of a human antibody [9]. Unfortunately, the interface changes between CDRs and new FRs may largely disturb the binding to the antigen. The initial CDR-grafted antibodies tend to lose the parental binding affinity, and therefore require additional work for back-mutation of several murine framework amino acids, which are regarded to be crucial for CDRs loop conformation [10]. Humanization *via* variable domain resurfacing is another approach that can maintain the specificity and binding affinity of parental antibody, which can reduce the immunogenicity of antibodies through the replacement of surface exposed residues in the murine FRs with those usually found in human antibodies [11], [12], [13], [14], [15]. Although the current molecular-biology techniques can render this approach more straightforward in practice, it is still difficult in determining the critical residues exposed in solvent on the surface, especially when requiring a reliable computer model of the antibody [14].

This study is in an attempt to humanize a murine anti-human TNF-α monoclonal antibody, m357, raised by immunization of mice with recombinant human TNF-α. In order to keep the parental binding affinity, we resurfaced m357 in the variable regions of both the heavy and light chains *via* computer-aided molecular modeling followed by functional characterization of the humanized antibody. The results indicated that the humanized antibody, h357, retained *in vitro* bioactivities similar to the parental antibody. Moreover, the humanized h357 IgG is capable of binding to the cells bearing the transmembrane TNF-α and triggering ADCC with effector cells and CDC. The humanized 357 IgG at a dose level of 50 µg/mouse/day significantly inhibited disease progression in a mouse model of collagen antibody-induced arthritis when compared with saline treated mice. These results provided a basis for the development of h357 IgG as therapeutic use.

## Results

### Molecular modeling of m357 variable fragments

**Figure 1:**
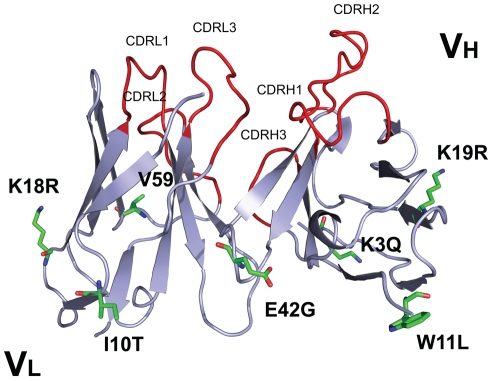
Molecular model of the m357 variable regions. The three-dimensional structure of m357 was generated by homology modeling using the crystal structures of the anti-breast tumor antibody SM3 (PDB code 1SM3) and the anti-Thermus aquaticus DNA polymerase I antibody (PDB code 1AY1) for the V_H_ and V_L_ domains, respectively. The six residues which are preferably mutated to “human” amino acid are indicated by stick structures (green). Four residues in the V_H_ framework and two residues in the V_L_ framework were humanized. Valine 59, which is within about 5 Å of the CDR2 in light chain and may affect the antigen binding, is retained for preserving the antigen binding affinity and shown as stick structure (orange). The CDR loops are shown in red color.

The murine monoclonal antibody derived from the hybridoma cell line, 357-101-4 (ECACC No. 92030603), is of therapeutic interest for its reported strong neutralizing biological activity against human TNF-α [16] The cDNAs encoding the heavy chain variable region (V_H_) and light chain variable region (V_L_) of the murine anti-human TNF-α monoclonal antibody (designated as m357) were first obtained by RT-PCR (**Figure S1**). The three-dimensional structure of the deduced amino acid sequences of the V_H_ and V_L_ fragments of m357 was constructed individually by homology modeling (program MODELLER) as described under “Materials and Methods”. Afterward, molecular models of the V_H_ and V_L_ domains of m357 were created using the crystal structures of the V_H_ domain of the anti-breast tumour antibody SM3 (PDB code 1SM3; 87% sequence identity) [17] and the V_L_ domain of the anti-Thermus aquaticus DNA polymerase I antibody (PDB code 1AY1; 85% sequence identity) [18], respectively. The final refined structures of the V_H_ and V_L_ domains of m357 were obtained through Discovery Studio modeling 2.1 program as shown in **Figure 1**.

### Humanization of m357 variable fragments

**Figure 2:**
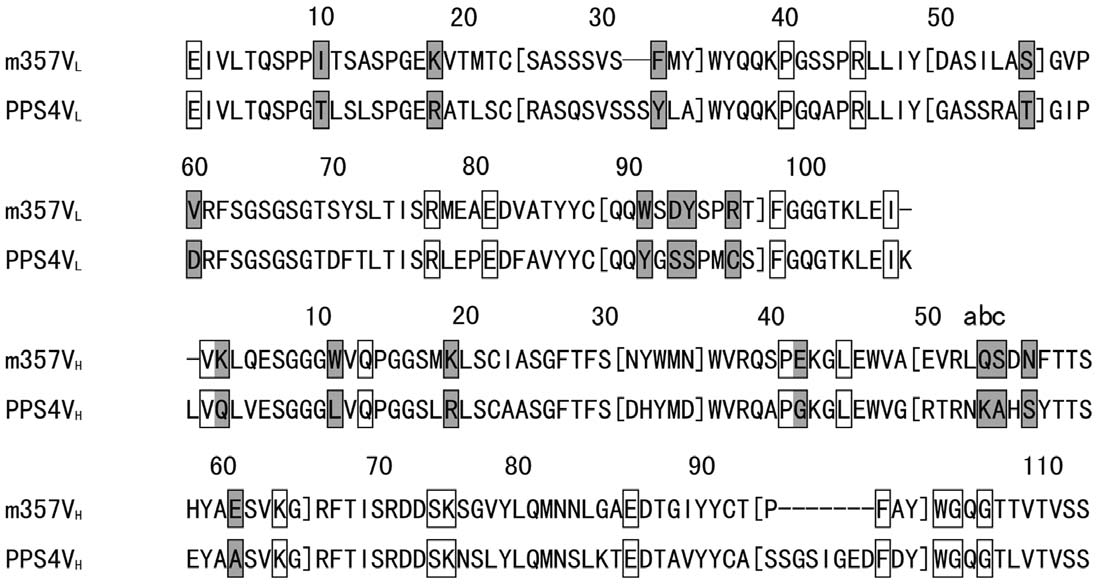
Amino acid sequence alignment of the V_H_ (A) and V_L_ (B) domains of m357 and PPS4. The sequences of PPS4 Fv which were the most homologous to m357 Fv used as the acceptor of human surface residues for m357 humanization is shown as PPS4V_L_ and PPS4V_H_ for comparison. CDR residues were within brackets. Conserved surface residues were marked with clear boxes, and non-conserved surface residues were marked with shadowed boxes. Amino acid sequences are numbered according to the Kabat's convention[33].

Humanization by variable domain resurfacing was first proposed by Padlan in 1991, in which the potential antigenic sites in the framework regions of an antibody were eliminated without affecting the antigen binding affinity [11]. This method is based on the hypothesis that the human anti-mouse antibody (HAMA) response to the variable region derives from the surface residues only and has adopted and modified by other researchers [14], [15], [19]. In this study, we performed the variable domain resurfacing of m357 with the following three steps: first, the molecular models of the V_H_ and V_L_ domains of m357 were constructed individually; second, we used AREAIMOL program to calculate the solvent accessible residues for identifying the non-human like framework surface residues, and finally we mutated these surface residues to the human counterparts according the results of the sequence alignment of human framework. In order to replace the non-human like framework surface residues of the murine m357 on the variable domain, a set of highly homologous surface residues from a human target sequence were selected. We searched the IMGT database (http://imgt.cines.fr/) to identify the human V_H_ and V_L_ sequence pairs, which are most homologous to the corresponding variable regions of m357, while eliminating the sequences of phage-display or humanized antibodies from our search results. The most identical surface residues found from human sequences were the variable regions of PPS4 (V_H_: 76% and V_L_: 73% in sequence identity in the FRs, respectively) (Genbank DQ187629 and DQ187550) (**Figure 2**). According to the calculation results of AREAIMOL program, 8 out of the 20 surface residues in the V_H_ were non-conserved between human and mouse sequences and 9 out of 16 surface residues were non-conserved in the V_L_. These 17 surface residues were the candidates to be replaced. However, the substitution of H52_B_Q, H52_C_S, H54N, H61E, L31F, L55S, L90W, L92D, L93Y and L96R in CDR regions may potentially alter the CDR's conformation, and V59, an additional non-conserved surface residue in light chain near the CDR2 within 5 Å, may potentially affect the binding affinity too. As a result, these 11 murine residues were retained for preserving the antigen binding affinity. Finally, the remaining 6 residues: H3K→Q, H11W→L, H19K→R, H42E→G, L10I→T and L18K→R were chosen to be replaced to the human conserved residues (**Figure 1**).

### Construction and expression of the humanized 357 IgG_1_

**Figure 3:**
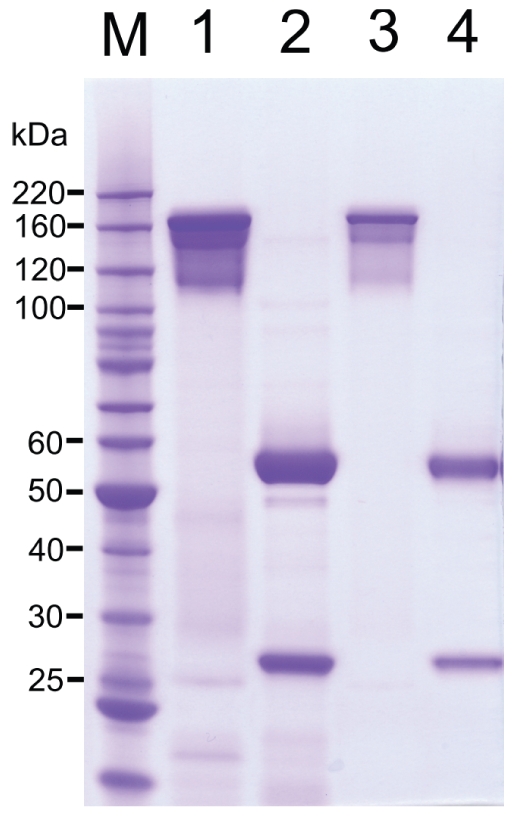
Purification of m357 and h357 antibodies. The indicated m357 (lanes 1 and 2) and h357 (lanes 3 and 4) were expressed in the mouse myeloma NS0 cells and purified from culture media by protein A column. The samples were electrophoresed on a 4∼12% SDS/Bis-Tris polyacrylamide gel with MOPS buffer under non-reducing conditions (lanes 1 and 3) and reducing conditions (lanes 2 and 4). Gel was stained with Coommassie blue. M, molecular mass standards.

The amino acid sequences of the humanized V_H_ and V_L_ of m357, comprising the signal peptide at the N-terminus, were in-frame fused to the human IgG γ1 heavy chain and kappa light chain constant regions, respectively. For the expression of an intact humanized 357 (h357) IgG_1_ molecule, two different mammalian expression vectors, pSecTag2/Hygro and pcDNA3.3, were used to adopt the humanized heavy chain and light chain of h357 IgG, respectively. After co-transfection and antibiotic selection, one of the clones showing the highest expression level was chosen for further studies. The expression level of the recombinant h357 IgG was ∼14 mg/L. Both m357 and h357 IgGs were purified by protein A chromatography individually and the protein purities were determined by SDS-PAGE. As shown in **Figure 3**, under non-reducing conditions, both antibodies showed a single band with a molecular mass of 155 kDa (lanes 1 and 3). Under reducing conditions, both antibodies yielded two protein bands with molecular masses of 55 kDa (heavy chain) and 26 kDa (light chain) (lanes 2 and 4).

### Neutralization of TNF-α-mediated cellular cytotoxicity by m357 and h357

**Figure 4:**
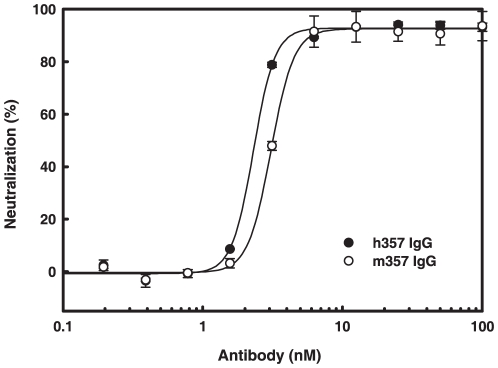
Neutralization of TNF-α-mediated cytotoxicity in L929 cells by m357 and h357 antibodies. Various concentrations of m357 (•) and h357 (○) antibodies were added to L929 cells cultured with 100 ng/ml of recombinant human TNF-α. The cells were incubated for 16 hrs at 37°C and cell viability was analyzed using a colorimetric MTT assay.

To examine the functional activity of the anti-TNF-α antibodies, the ability of the antibody to inhibit soluble TNF-α activity was performed. TNF-α causes cell cytotoxicity to murine L929 cells. Both m357 and h357 IgGs were evaluated in L929 assays by co-incubation of antibodies with recombinant human TNF-α. As shown in **Figure 4**, TNF-α-mediated cytotoxicity in L929 cells treated with 100 ng/ml of human TNF-α was effectively neutralized by both m357 and h357 IgGs in a dose dependent manner, with ED_50_ of 3.07 nM and 2.30 nM, respectively. The results indicated that the humanized 357 IgG retained the TNF-α neutralization activity at concentrations similar to that of the murine IgG.

### Binding affinities of h357 IgG to soluble and membrane form TNF-α

**Figure 5:**
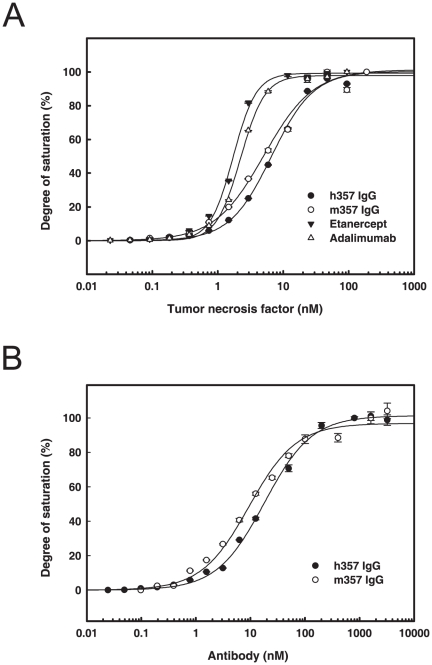
Binding affinities of h357 IgG to soluble and membrane form TNF-α. (A) Binding affinities of different TNF-α anatagonists to soluble TNF-α. Microtiter plates with the anti-human IgG Fcγ fragment captured h357 (•), etanercept (▾) and adalimumab (△) and the anti-mouse IgG Fcγ fragment captured m357 (○) were incubated with various concentrations of soluble TNF-α for 1 hr at 37°C. After washing, the bound TNF-α was detected by a mouse monoclonal anti-TNF-α antibody conjugated with horseradish peroxidase. Absorbance was read at 450 nm on a microplate reader. (B) The saturation binding curves of m357 and h357 IgGs to transmembrane TNF-α. Transmembrane TNF-α-transfected NS0 cells were incubated with serial log dilutions of m357 (○) or h357 (•) antibody for 1 hr at 4°C. Cell were washed and incubated with Alexa Fluor 488 goat anti-mouse IgG (H+L) for m357 and Alexa Fluor 647 goat anti-human IgG (H+L) for h357, respectively for 1 hr at 4°C. Cells were then washed and analyzed by FACSCalibur flow cytometer.

A higher binding affinity between antagonists and soluble TNF-α can help maintain the stability of neutralizing complex and become a critical factor for neutralizing activity of anti-TNF-α antibodies [2]. The relative binding affinities of h357, m357 IgGs and two marketed TNF-α antagonists, etanercept and adalimumab, to soluble form TNF-α were compared by ELISA binding assays. As shown in **Figure 5A**, the binding affinities of h357, m357, etanercept and adalimumab for soluble human TNF-α were all in the nanomolar range, with K_D_ values of approximately 7.8 nM, 5.5 nM, 2.2 nM and 2.9 nM, respectively. The similar K_D_ values in h357 and m357 IgGs indicated that the humanization process did not alter the TNF-α binding strength of the parental mouse antibody.

TNF-α exists as a membrane associated precursor (transmembrane TNF-α) from which the mature soluble form is released by proteolytic cleavage mediated by TNF-α converting enzyme. Many studies have investigated that TNF-α antagonists can cause cell lysis by apoptotic, ADCC, CDC and outside-to-inside signaling mechanisms given that it has the binding ability to transmembrane TNF-α [1], [20], [21], [22]. To analyze the transmembrane TNF-α binding ability of h357, we transfected an uncleavable form of transmembrane TNF-α cDNA into NS0 cells to express it on the cell surface, and used flow cytometry to assess the binding activities of both m357 and h357 IgGs. The data in **Figure 5B** indicated that both m357 and h357 IgGs can bind to transmembrane TNF-α in a concentration-dependent manner, with K_D_ of 12.0 nM and 16.8 nM, respectively. The similar K_D_ values in m357 and h357 indicated that the humanization process did not alter the transmembrane TNF-α binding affinity. The binding affinity in the nanomolar range suggested h357 IgG could potentially trigger more effector functions or apoptosis mechanism through binding to transmembrane TNF-α.

### Ability of h357 and m357 to mediate ADCC and CDC

**Figure 6:**
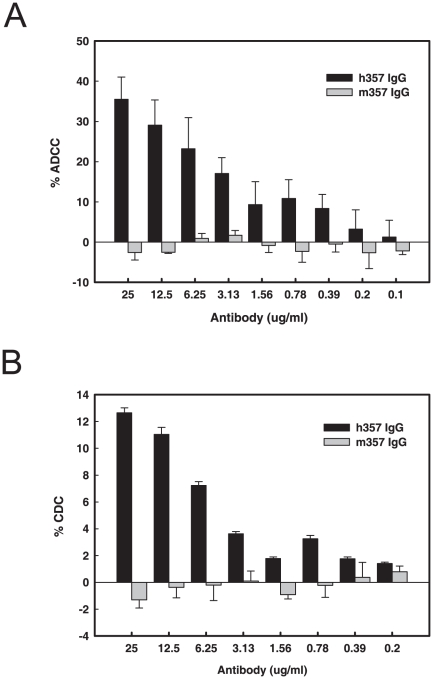
ADCC and CDC of h357 and m357 IgGs against transmembrane TNF-α-expressing cells. Transmembrane TNF-α-expressing NS0 cells were incubated in the presence of different concentrations of h357 or m357 antibody for 1 hr. Subsequently, PBMCs for ADCC or human complement-rich serum for CDC were used as effector cells and the source of complement, respectively, and transmembrane TNF-α-expressing cells were used as targets. The cytotoxicity was calculated by measuring the amount of LDH released from the cytosol into the supernatant. Results are presented as percent cell lysis in antibody treated groups compared to 100% lysis in lysis buffer treated cell group.

Previous studies have been reported that the binding affinity of infliximab and adalimumab toward the membrane form of TNF-α were better than that of etanercept, which can affect the cell killing of cell surface-expressed TNF-α by ADCC, CDC or apoptosis mechanisms and this may be one of reasons that cause different effects on clinical diseases [3]. Cell surface-expressed TNF-α like macrophages and monocytes play a critical role in the Granulomatous diseases such as Crohn's disease and Wegener's granulomatosis, and the cells can be killed directly by ADCC or CDC effects [23]. When TNF-α antagonists bind to cells expressing the transmembrane form of TNF-α, these cells will be targeted by natural killer cells or triggered the activation of systemic complement. The presence of the Fc region of human IgG1 in h357 may potentially cause cell lysis in TNF-α-producing cells through effector functions. The ability of h357 and m357 IgGs to mediate ADCC and CDC against the cells expressing the transmembrane form of TNF-α were conducted. In ADCC assay, more than 20% of the TNF-α bearing NS0 target cells were lysed by h357 IgG at 6.25 µg/ml at a 20∶1 effector to target (E:T) ratio (**Figure 6A**). In CDC assay, h357 IgG was also capable in lysing transmembrane TNF-α cells in the presence of human complement (**Figure 6B**). In contrast, m357 IgG exhibits neither ADCC nor complement-mediated lysis because the human natural killer cells or systemic complement are unable to recognize the mouse Fc fragment (**Figure 6**). These data indicated that h357 IgG can mediate ADCC and CDC effects upon binding to the transmembrane TNF-α expressed on cell surface and therefore it possesses the potential to be developed into a more effective TNF-α-neutralizing antibody similar to those therapeutic antibodies with ADCC and CDC capabilities.

### *In vivo* inhibition of murine collagen antibody-induced arthritis with h357 IgG

**Figure 7:**
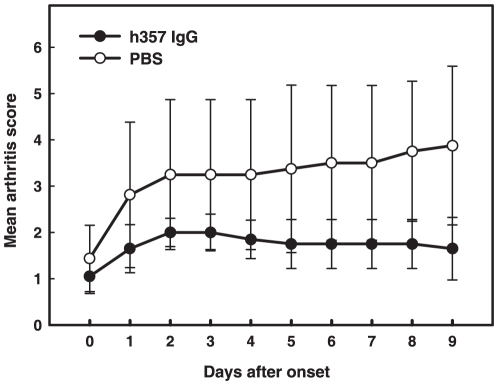
Assessment of scores in collagen antibody-induced arthritic mice treated with h357 IgG and phosphate buffered saline. At the time of disease onset (day 0), mice were given by intra-peritoneal injection of h357 IgG (50 µg) or 100 µl of phosphate buffered saline (non-treatment) once-daily for 9 consecutive days. The arthritis scores were compared between non-treated (n = 4) and h357 IgG (n = 5) treated groups during the course of treatment. Scoring results were expressed as mean ± standard deviation (SD). When mice were treated with h357 IgG, the rate of arthritis development was significantly reduced (P<0.05).

To assess the therapeutic effects of h357 on rheumatoid arthritis development, the collagen antibody-induced arthritis mouse model was adopted, in which the disease was induced by the systemic administration of a cocktail of monoclonal antibodies that target various regions of collagen type II, which is one of the major constituents of articular cartilage matrix proteins, followed by lipopolysaccharides [24]. The mice were intraperitoneally injected with h357 IgG (50 µg/mouse) or phosphate buffered saline (non-treatment group) once-daily for 9 consecutive days. As shown in **Figure 7**, progressed to significantly less severe disease than the saline-treated mice group (P<0.05).

## Discussion

TNF-α antagonists, including antibodies, have been developed for binding to TNF-α as a mean to inhibit its biological activities, including TNF-α-induced cell activation and cytotoxicity. Anti-TNF-α therapies have been applied to a variety of autoimmune disorders, such as rheumatoid arthritis [25], inflammatory bowel disease [26] and systemic lupus erythematosus [27]. A common feature of these therapeutic antibodies is that they all have the capacity to neutralize soluble form of TNF-α activity effectively with high binding affinity in the range of nM or less. Meanwhile, antibodies with slow dissociation kinetics and good pharmacokinetics are also required since TNF-α is secreted constitutively from the activated macrophages, monocytes, and lymphocytes from patients with the above chronic inflammatory diseases. In this study, we adopted a reported murine anti-human TNF-α antibody with strong neutralizing activity against TNF-α [16] for humanization by resurfacing approach. The humanized h357 IgG retained the high antigen binding affinity and bioactivity as compared with the parental murine IgG based on TNF-α binding and neutralization assays, respectively. We also compared the relative TNF-α binding affinity of h357 IgG with two anti-TNF-α agents, the monoclonal anti-TNF-α antibody adalimumab and the soluble TNF receptor fusion etanercept. Although the relative binding affinity of h357 IgG is slightly lower than both etanercept and adalimumab, the relative TNF-α neutralization activities were found to be similar (data not shown). It is possible that the binding kinetics (association and dissociation rates) among these anti-TNF-α agents are different. Further studies in the binding kinetics of h357 IgG need to be conducted to elucidate the correlation between binding affinity and bioactivity. In the mean time, affinity maturation by CDR-shuffling using phage display approaches may need to be adopted if further affinity improvement of h357 IgG is required for therapeutic use.

Protein therapeutics from exogenous sources can potentially elicit immune response when administered in humans. The anti-drug antibodies could lead to partially or completely loss of drug efficacy. Preclinical immunogenicity assessment has drawn a lot of attention to FDA and is highly encouraged in the preclinical development stages. Many techniques, such as humanization, deimmunization and deaggrgation have been adopted to alter the structure of a protein in order to reduce the potential risk of immugenecity. Antibody humanization by CDR-grafting can often result in decrease or completely loss of antigen binding affinity because the murine frame-work regions encompassing the variable regions often contribute the integrity of antibody structure for the formation of correct CDR-epitope complex. The deimmunization approach we have adopted is a three dimensional structure resurfacing method based on the fact that most antigenic response to the variable region is directed to surface residues only. By replacing the amino acids that are potentially antigenic whereas preserving the most murine primary structure in the variable regions should in theory minimize the conformational change of the parental murine antibody and thus retain the antigen binding affinity. So far, there is little information available about the effects of antibody deimmunization by resurfacing on the antigen binding affinities. In this study, we demonstrated that this approach is feasible in retaining the antibody-antigen affinity. The next question is whether this resurfacing approach is truly a way to generate non-immunogenic proteins. Since antibody drugs are highly species specific, it is difficult to assess the immune response in humans when conducting preclinical animal experiments even with non-human primates. The adopted three dimensional structure resurfacing method currently we used is distinct from the *in silico* primary structure-based algorisms which mainly focused on MHC class II peptide binding predictions. Meanwhile, recently developed T-cell based procedures are showing promise for predicting potential immunogenicity with some biologics including monoclonal antibodies. Further experimental work focusing on both *in silico* and T-cell assays of the h357 IgG will be assessed to further minimize the immunogenicity issues for drug development.

## Materials and Methods

### Ethics Statement

Human whole blood was collected from healthy donors at Hsinchui Blood Center, Taiwan, under a protocol approved by the Institutional Review Board of Industrial Technology Research Institute (IRB number 9901008) and reviewed by the Joint Institutional Review Board (http://www.jirb.org.tw/), case number 10-S-004, a committee for allowing all blood centers in Taiwan to provided blood for research purposes from those donors with written consent. All animal work was reviewed and approved by the Institutional Animal Care and Use Committee of Industrial Technology Research Institute (approval number ITRI-IACUC-2010-030R).

### Computer modeling of m357 for resurfacing

The homology modeling process of m357 was performed using Discovery Studio Modeling 2.1 (Accelrys, Inc., San Diego, CA). Two separate BLASTP searches were performed for V_L_ and V_H_ of m357. For identifying the homology of m357 protein sequences, sequences were analyzed by searching over the Protein Data Bank (PDB) (http://www.rcsb.org/pdb/home/home.do). The three-dimensional (3D) structure of the m357 Fv fragment was constructed by homology modeling based on the structures of the V_H_ domain of the murine anti-breast tumor antibody Fab fragment SM-3[PDB entry: 1SM3] [17] and the V_L_ domain structure of an anti-thermus aquaticus DNA polymerase I monoclonal antibody Fab fragment [PDB entry: 1AY1] [18]. The final 3D model was generated by MODELLER module [28], which implements an automated approach to comparative protein structure modeling by satisfaction of spatial restraints. It performed automatically protein homology modeling and loop modeling for m357. Model building of the CDR loops were performed by selecting template structures from the PDB database with the highest sequence identity using Model antibody Loops module and were refined using Loop Refinement module in order to minimize steric clashes and ensure correct bond lengths and angles. Then, this whole model could be further refined by CHARMm [29] with Accelrys CHARMm forcefield in Discovery Studio Modeling 2.1. The structure was energy-minimized in two steps, first by 5000 steps of restrained steepest descent minimization, and followed by another 5000 steps of conjugated gradient minimization while the alpha carbons of the framework were held fixed in position. The solvent-accessible surface areas of m357 residues were calculated on the three-dimensional model by the AREAIMOL program [30]. The residues which relative accessibility was greater than 30% were defined as being accessible.

### Construction, expression and purification of humanized anti-human TNF-α IgG

The total RNA of around 10^7^ cells of the hybridoma 357-101-4 (ECACC No. 92030603) which secrete anti-human TNF-α IgG (m357) was isolated and the first-strand cDNA was generated using oligo (dT) primers with Superscript III reverse transcriptase (Invitrogen, San Diego, CA). The cDNA fragments encoding the heavy chain and light chain variable regions of m357 were obtained by PCR with the Heavy Primers (Cat. No. 27-1586-01, GE Healthcare) and the Light Primer Mix (Cat. No. 27-1583-01, GE Healthcare), respectively. The cDNA fragments encoding the humanized heavy chain and light chain variable regions of m357 were obtained by over-lapping PCR with primers covering the humanized regions. For the construction of the heavy chain of the humanized 357 (h357) antibody, the cDNA construct consisting the murine Ig kappa-chain V-J2-C signal peptide derived from the mammalian expression vector pSecTag2/Hygro (Invitrogen, San Diego, CA), the V_H_ region of h357, and the human IgG1 constant region (CH1, hinge, CH2 and CH3) which derived from the cloning vector pFUSE-CHIg-hG1 (Invivogen, San Diego, CA) were obtained by over-lapping PCR, followed by sub-cloning into the vector pSecTag2/Hygro (Invitrogen, San Diego, CA) at Nhe I and Not I sites. For the construction of the light chain of the humanized 357 (h357), the cDNA construct consisting the above murine Ig kappa-chain V-J2-C signal peptide, the V_L_ region of h357, and the human kappa light chain constant region which derived from the cloning vector pFUSE2-CLIg-hk (Invivogen, San Diego, CA) were obtained by over-lapping PCR, followed by sub-cloning into the mammalian expression vectors pcDNA3.3-TOPO TA (Invitrogen, San Diego, CA). Plasmids containing the heavy and light chain cDNAs of h357 IgG_1_ were co-transfected into mouse myeloma NS0 cells (European Collection of Animal Cell cultures, Salisbury, Wiltshire, UK) using Effectene (Qiagen). After selection with hygromycin B (400 µg/ml) and G418 (800 µg/ml) for 4 weeks, culture media from several stable clones were screened by ELISA with human TNF-α as coating and anti-human IgG Fcγ-HRP as detection reagents. An h357 IgG_1_ high-expressing clone was picked and cultured in shaker flask at an initial seeding density of 5×10^5^ cells/ml in a chemically-defined medium HyQNS0 (Hyclone) without serum addition. Media were harvested 5 days later and h357 IgG_1_ was purified from the supernatant by Protein A (GE Healthcare) chromatography.

### Anti-TNF-α neutralization potency assay

Neutralizing activities of m357 and h357 against human TNF-α were measured on the murine fibroblast L929 cells (ATCC Cat. No. CCL-1) treated with actinomycin D according to the method described previously[31]. Briefly, L929 cells were seeded in triplicate at 3×10^5^ cells/well into a 96-well plate and cultured in RPMI 1640 medium supplemented with 10% (v/v) fetal bovine serum for 16 hours. Then, several dilutions of the antibodies were prepared in medium containing actinomycin D (2 µg/ml) and TNF-α (100 ng/ml) and incubated at 37°C for 16 hours. After the supernatant were removed, 3-4,5-dimethylthiazol-2-yl-2,5-diphenyltetrazolium bromide (MTT) (5 mg/ml) (Sigma-Aldrich) was added and incubated in 37°C for 4 hours. SDS solution (10%) was then added to the well. After 24 hours of incubation at room temperature, color in each well was recorded by colorimeter at 570 nm. Blank control (culture alone), TNF-α control (TNF-α alone), and antibody control (antibody alone) were also designed in the experiment. The ED_50_ value was calculated by complex sigmoid non-linear regression analysis using SigmaPlot software (Systat software, Inc. Richmond, CA).

### Stable expression of transmembrane TNF-α on NS0 cells

A mutant form of transmembrane TNF-α, which is resistant to TNF-α converting enzyme-mediated cleavage, was generated by site-directed mutagenesis as described previously [32]. In this uncleavable form of transmembrane TNF-α, the amino acids +1 through +12 of the native transmembrane TNF-α were deleted. An uncleavable form of transmembrane TNF-α gene was cloned into pSecTag2/Hygro mammalian expression vector (Invitrogen, San Diego, CA) and transfected into mouse myeloma NS0 cells by Effectene for expressing transmembrane TNF-α on cell surface.

### Antibody binding studies

The binding activities of h357 IgG, m357 IgG, etanercept and adalimumab to soluble TNF-α were determined by ELISA. For capturing h357 IgG, etanercept (Wyeth) and adalimumab (Abbott), the microtiter plate was coated with 2 µg/ml of goat anti-hunam IgG Fcγ fragment (Jackson ImmunoResearch Laboratories, Inc., West Grove, PA) in PBS overnight at 4°C. For capturing m357 IgG, the microtiter plate was coated with goat anti-mouse IgG Fcγ fragment (Jackson ImmunoResearch Laboratories, Inc., West Grove, PA). After blocking the wells with StartingBlock™ blocking buffer (Thermo Scientific), 2 µg/ml each of the TNF-α antagonists, as mentioned above, were added and incubated for 1 hr at 37°C. After washing, varing concentrations of human recombinant TNF-α (eBioscience, Inc., San Diego, CA) in PBS were incubated for 1 hr at 37°C. After washing, the bound soluble TNF-α was detected by incubation with horseradish peroxidase-conjuagted mouse monoclonal anti-TNF-α antibody (clone F6C5, Abcam, Cambridge, MA) for 1 hr at 37°C and using 3,3′,5,5′,-tetramethylbenzidine as substrate. Absorbance was read at 450 nm on a microplate reader. The dissociation constant (K_D_) of each antagonist was calculated as the concentration of TNF-α required to achieve half-saturation of the total binding sites (maximum absorbance).

### Saturation binding assay of m357 and h357 IgGs to transmembrane TNF-α

NS0 cells stably expressing the transmembrane TNF-α were incubated with serial log dilutions of m357 and h357 IgGs for 1 hr at 4°C in phosphate buffered saline (PBS) containing 2% fetal bovine serum (fluorescence-activated cell sorting [FACS] buffer). Cells were washed with a FACS buffer for 3 times and were then stained with Alexa Fluor 488 conjugated goat anti-mouse IgG (H+L) (Invitrogen, San Diego, CA) for m357 IgG and Alexa Fluor 647 conjugated goat anti-human IgG (H+L) (Invitrogen, San Diego, CA) for h357 IgG, respectively for 1 hr at 4°C. Fluorescence intensities were measured using a FACSCalibur flow cytometer (Becton Dickinson, San Jose, CA).

### ADCC and CDC assays

Peripheral blood mononuclear cells (PBMCs) were prepared by Ficoll-Hypaque density gradient centrifugation. ADCC and CDC activities of h357 and m357 IgGs were measured by LDH Cytotoxicity Detection Kit (Clontech), which measures lactate dehydrogenase (LDH) activity released from the cytosol of damaged cells. Briefly, NS0 cells stably expressing the transmembrane TNF-α were incubated with different concentration of h357 or m357 IgG for 1 hr in assay medium (DMEM +1% FBS) in a 5% CO_2_ incubator at 37°C, followed by the addition of either human peripheral blood mononuclear cells (PBMC) as effector cells (effector to target ratio  = 20∶1 for ADCC assay) or human complement-rich serum (Quidel) (1.25% vol/vol, for CDC assay). After an additional incubation for 16 hrs for ADCC assay and 5 hrs for CDC assay at 37°C, respectively, 100 µl of supernatant from each well were transferred into a clean flat-bottom 96-well plate. LDH substrate (100 µl) was added to each well and incubated for 30 min at room temperature protected from light. The absorbance of the samples was measure at 490 nm with an ELISA reader. Maximum LDH release was determined by incubating cells with lysis buffer. Percentage of specific lysis was calculated according to the following formula: % cytotoxicity  = [experimental release – spontaneous release]/[maximum release – spontaneous release]×100.

### *In vivo* inhibition of murine collagen antibody-induced arthritis with h357 IgG

For collagen antibody-induced arthritis experiments, 8- to10-week old male DBA/1J mice were purchased from Jackson laboratories (Bar Harbor, ME). Mice were maintained under a climate controlled environment in a 12-h light/dark cycle. Arthritic mice were induced by intra-peritoneal (i.p.) injection of 3 mg/mouse of type II collagen specific antibodies (ModiQuest Research). Mice were further boosted with 25 µg lipopolysaccharides (LPS) (Sigma) by i.p. injection on day 6. Clinical arthritis scores were evaluated using a scale of 0–2 for each paw for a total score of 8. Paws were assigned a clinical score based on the index (ModiQuest Research scoring method): 0 =  normal; 0.25 =  one or two swollen toes; 0.5 =  three and four swollen toes; 0.75 =  slightly swollen footpad or ankle; 1 =  swollen footpad or ankle; 1.25 =  one or two swollen toes and swollen footpad or ankle; 2.0 =  swollen toes and swollen footpad and swollen ankle. At the time of disease onset, mice were administered by i.p. injection of 50 µg per does for 9 consecutive days. Scoring results were expressed as mean ± standard deviation (SD). Statistical differences between the control (phosphate buffered saline) and experimental groups were analyzed by Mann-Whitney U test for the severity of arthritis.

## Supporting Information

Figure S1 **RT-PCR results showing the variable fragments and schematic representation of the strategy to assemble the heavy and light chains.** (A) Amplification of the variable fragment cDNAs from the mouse hybridoma 357-101-4 secreting m357 IgG by RT-PCR with the Heavy Primers (Cat. No. 27-1586-01, GE Healthcare) and the Light Primer Mix (Cat. No. 27-1583-01, GE Healthcare), respectively. Lane M: Molecular weight marker. Lane 1: V_H_, ∼340 bp. Lane 2: V_L_, ∼325 bp. (B) Assembly of the cDNAs encoding the open reading frames of the heavy and light chains of the humanized 357 (h357) IgG1 by over-lapping PCR, respectively. The cDNA construct consisting the murine signal peptide (SP) derived from the cloning vector pSecTag2/Hygro, the V_H_ region of h357, and the human IgG1 constant region (CH1, hinge, CH2 and CH3) derived from the cloning vector pFUSE-CHIg-hG1 were obtained by over-lapping PCR, followed by sub-cloning into the vector pSecTag2/Hygro at Nhe I and Not I sites. The cDNA construct consisting the above signal peptide, the V_L_ region of h357, and the human kappa light chain constant region derived from the cloning vector pFUSE2-CLIg-hk were obtained by over-lapping PCR, followed by sub-cloning into the mammalian expression vectors pcDNA3.3-TOPO TA (Invitrogen, San Diego, CA). The locations of the primers and the restriction sites are shown in the diagram. SP, murine Ig kappa-chain V-J2-C signal peptide.
(EPS)
